# SOX2 Regulates Neuronal Differentiation of the Suprachiasmatic Nucleus

**DOI:** 10.3390/ijms23010229

**Published:** 2021-12-26

**Authors:** Arthur H. Cheng, Samuel W. Fung, Sara Hegazi, Osama Hasan Mustafa Hasan Abdalla, Hai-Ying Mary Cheng

**Affiliations:** 1 Department of Biology, University of Toronto Mississauga, Mississauga, ON L5L 1C6, Canada; ahh.cheng@mail.utoronto.ca (A.H.C.); samuel.fung@mail.utoronto.ca (S.W.F.); sara.hegazi@mail.utoronto.ca (S.H.); osama.abdalla@mail.utoronto.ca (O.H.M.H.A.); 2 Department of Cell & Systems Biology, University of Toronto, Toronto, ON M5S 3G5, Canada

**Keywords:** suprachiasmatic nucleus, neuronal differentiation, development, circadian clock

## Abstract

In mammals, the hypothalamic suprachiasmatic nucleus (SCN) functions as the central circadian pacemaker, orchestrating behavioral and physiological rhythms in alignment to the environmental light/dark cycle. The neurons that comprise the SCN are anatomically and functionally heterogeneous, but despite their physiological importance, little is known about the pathways that guide their specification and differentiation. Here, we report that the stem/progenitor cell transcription factor, *Sex determining region Y-box 2* (*Sox2*), is required in the embryonic SCN to control the expression of SCN-enriched neuropeptides and transcription factors. Ablation of *Sox2* in the developing SCN leads to downregulation of circadian neuropeptides as early as embryonic day (E) 15.5, followed by a decrease in the expression of two transcription factors involved in SCN development, *Lhx1* and *Six6*, in neonates. Thymidine analog-retention assays revealed that *Sox2* deficiency contributed to reduced survival of SCN neurons during the postnatal period of cell clearance, but did not affect progenitor cell proliferation or SCN specification. Our results identify SOX2 as an essential transcription factor for the proper differentiation and survival of neurons within the developing SCN.

## 1. Introduction

Many organisms have evolved endogenous circadian rhythms to anticipate challenges and opportunities arising from the Earth’s daily cycles of light and darkness. In mammals, circadian rhythms are orchestrated by the suprachiasmatic nucleus (SCN), a bilateral structure located in the anterior hypothalamus. Unlike most other tissues, the SCN is an autonomous circadian pacemaker that can exhibit robust rhythmic oscillations for weeks, even when isolated and maintained ex vivo as organotypic slice cultures [1]. Dissociated SCN neuronal cultures further demonstrated that each neuron in the SCN network harbors a molecular clock machinery and is capable of independent cellular oscillations [2].

At the molecular level, circadian rhythms are generated by a series of transcription-translation feedback loops (TTFLs) through a negative feedback mechanism. In the primary feedback loop, heterodimers of the transcription factors, CLOCK and BMAL1, drive the expression of core clock genes including *Per* and *Cry* through recognition of E-box elements within gene promoters [3,4,5,6,7]. Once translated, PER:CRY heterodimers inhibit CLOCK:BMAL1, thus repressing their own transcription. Eventual degradation of PER and CRY proteins results in the derepression of E-box-mediated transcription, permitting the initiation of a new round of gene expression [3,4,8,9,10,11]. In addition to the TTFLs, individual SCN neurons synthesize neuropeptides and neurotransmitters to coordinate endogenous oscillations and entrainment at the tissue level [12,13,14]. As the structure responsible for receiving and integrating time cues from the environment and for driving tissue-specific circadian oscillators through humoral and endocrine signaling, the SCN is regarded as the master circadian clock that dictates the rhythm of the organism [15,16].

The murine SCN is comprised of approximately 20,000 neurons, in addition to astrocytes, oligodendrocytes, endothelial cells, microglia, and polydendrocytes [17,18]. Neurons of the SCN can be further categorized into subtypes based on spatial distribution, circadian rhythmicity, light responsiveness, and the expression pattern of specific cellular markers including neuropeptides [17,19,20,21,22]. Given the heterogeneity of the SCN, its genesis and maturation are believed to be controlled by intricate developmental pathways. Transcription factors such as *Six3*, *Six6*, *Lhx2*, *Vax1*, and *Foxd1* have been shown to be necessary for SCN specification [23,24,25,26,27]. However, to date, only one transcription factor, *Lhx1*, has been demonstrated to regulate SCN differentiation and the expression of SCN neuropeptides [28]. To advance our understanding of SCN development, we focused our attention on the transcription factor, SRY (sex-determining region Y)-box 2 (SOX2).

SOX2 is first expressed in the inner cell mass and the trophectoderm of the blastocyst, and is present in stem cells of different tissues during later developmental stages [29]. In the brain, SOX2 is highly expressed in neural progenitor cells, which constitute the ventricular zone of the developing neural tube [29]. As cells differentiate and move out of the neurogenic ventricular zone of the neural tube, they turn off the expression of SOX2 [29]. However, a few groups of post-mitotic, differentiated cells in the adult brain, including SCN neurons, retain robust SOX2 expression [30,31]. Previous studies have found that constitutive or conditional ablation of *Sox2* results in varying degrees of developmental defects, depending on the timing and location of *Sox2* depletion. *Sox2* ablation at E9.5 in *Foxg1*-expressing cells leads to severe developmental defects in the telencephalon, including loss of the medial ganglionic eminence and olfactory neuroepithelium [32]. Deletion of *Sox2* in *Nestin*-expressing cells results in the failure to maintain hippocampal radial glia and neurogenesis, culminating in the loss of the hippocampal dentate gyrus [33]. *Sox2* hypomorphic mice have reduced GABAergic interneurons in the newborn cortex and adult olfactory bulb, accompanied by increased neurodegeneration and impaired adult neurogenesis in the subventricular and subgranular zones [34,35]. These mice also exhibit reduced pituitary size and hormone production as well as testicular atrophy and infertility with age [36].

Using *Vgat-cre* transgenic mice to ablate *Sox2* expression in the SCN, we recently highlighted the key roles of SOX2 in regulating SCN circadian timekeeping [37,38]. *Sox2* deletion impairs light-induced entrainment and the generation of robust, precise, and consolidated behavioral rhythms. RNA-seq analysis revealed marked changes in the circadian transcriptome of the adult SCN in the absence of SOX2; in particular, we noted significant downregulation of genes encoding core clock components, neuropeptides, neuropeptide receptors, and transcription factors with documented roles in development. Some of these transcriptional perturbations are likely to be through a direct interaction between SOX2 and the promoter or enhancer of the target gene in the adult SCN, as was demonstrated for the *Period2* gene. However, there may also be indirect mechanisms stemming from a potential impact of *Sox2* ablation on SCN development. Given that the SCN of *Vgat*-specific *Sox2* knockout mice was visibly smaller than *Sox2^fl/fl^* littermates [37], it is plausible that *Sox2* deletion disrupted the development and/or maturation of the mutant SCN.

Here, we show that SOX2 is indispensable for the proper regulation of SCN neuronal differentiation and survival. We demonstrate that *Vgat*-driven Cre recombinase activity ablates SOX2 expression in the SCN at or before the onset of SCN neurogenesis. The absence of SOX2 does not impair cell proliferation during SCN neurogenesis, but instead leads to a significant reduction in the expression of arginine vasopressin (*Avp*) and vasoactive intestinal peptide (*Vip*) and the number of *Avp^+^* and *Vip^+^* cells in the SCN as early as E15.5. Notably, the downregulation of these neuropeptides precedes the decrease in expression of *Six6* and *Lhx1*. Other SCN-enriched neuropeptides such as prokineticin 2 (*Prok2*) and neuromedin S (*Nms*) are also downregulated in the neonatal, *Sox2*-ablated SCN, whereas transcription factors previously implicated in SCN specification and differentiation, including *Six3*, *Vax1*, *Rora*, and *Rorb*, are not affected. Moreover, the absence of SOX2 increases the numbers of SCN neurons that are normally lost during the early postnatal period. Taken together, our findings identify a critical role of SOX2 in the differentiation of SCN neurons, a role that appears to be functionally independent of *Six6, Lhx1,* and other transcription factors that have been implicated in SCN development.

## 2. Results

### 2.1. Sox2 Ablation in the Vgat-cre;Sox2^fl/fl^ Model Occurs at or before E12.5 and Is Restricted to the SCN

**Figure 1:**
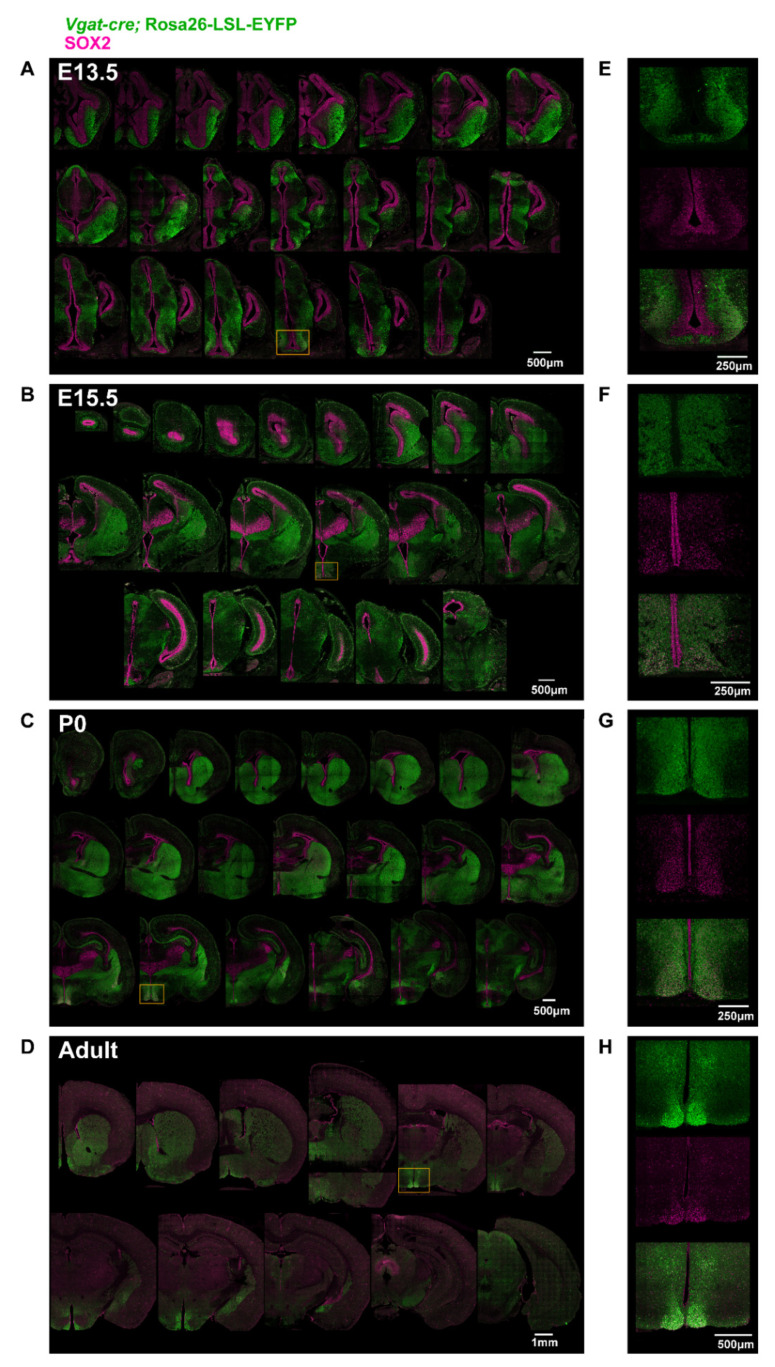
Expression pattern of VGAT and SOX2 in the developing and adult mouse brain. (**A**–**H**) Representative photomicrographs of EYFP (proxy for VGAT; green) and SOX2 (magenta) expression in the brains of *Vgat-cre;Rosa26-LSL-EYFP* mice at (**A**,**E**) E13.5, (**B**,**F**) E15.5, (**C**,**G**) P0, and (**D**,**H**) the adult stage along the rostral-caudal axis (**A**–**D**, from top left to bottom right). Orange rectangles highlight the hypothalamic region containing the developing or mature SCN, which is given in higher magnification in (**E**–**H**).

**Figure 2:**
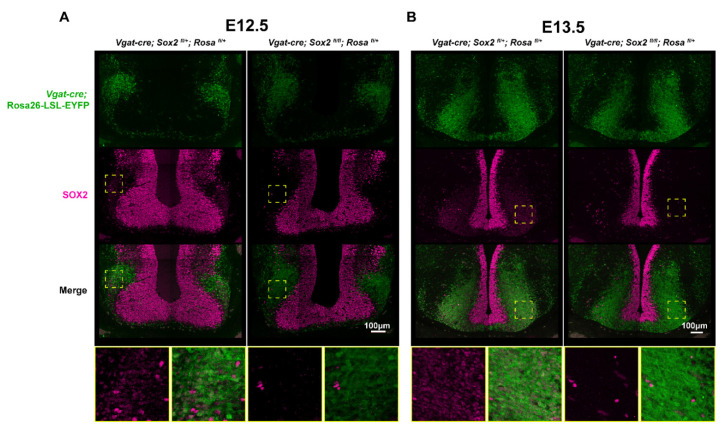
Cre-mediated excision of *Sox2* occurs at or before E12.5 in the *Vgat-cre;Sox2^fl/fl^* model. (**A**,**B**) Expression profile of EYFP (proxy for VGAT, green) and SOX2 (magenta) in the ventral anterior hypothalamus of *Sox2* heterozygous (*Vgat-cre;Sox2^fl/+^;Rosa^fl/+^*) and *Sox2* cKO (*Vgat-cre;Sox2^fl/fl^;Rosa^fl/+^*) mice at (**A**) E12.5 and (**B**) E13.5. Yellow dotted squares highlight the parenchymal region with colocalized expression of VGAT and SOX2 in control animals, and the corresponding anatomical location in mutants. Higher magnification images of the outlined regions are shown in the bottom-most panels. One litter of E12.5 *Sox2* heterozygous (*n* = 2) and *Sox2* cKO (*n* = 2) animals were analyzed. Two litters of E13.5 *Sox2* heterozygous (*n* = 1 from each litter; total *n* = 2) and *Sox2* cKO (*n* = 1 from each litter, total *n* = 2) animals were analyzed.

To determine when *Sox2* gene excision occurs in the SCN of *Vgat-cre;Sox2^fl/fl^* mice, we mapped the activity of Cre recombinase by breeding *Vgat-cre* animals with the *Rosa26-loxP-STOP-loxP-EYFP* (*Rosa^fl/fl^*) strain, the latter expressing enhanced yellow fluorescent protein (EYFP) upon excision of the loxP-flanked STOP cassette [39]. The expression of EYFP was co-mapped with SOX2 in the brains of E13.5, E15.5, P0, and adult *Vgat-cre;Rosa^fl/+^* mice (Figure 1A–H). Strong EYFP and SOX2 expression was detected by immunofluorescence (IF) in the ventral anterior hypothalamus of E13.5 embryos, and in E15.5, P0, and adult SCN (Figure 1E–H). Noticeably, from E13.5 onwards, EYFP and SOX2 expression was highly colocalized in the SCN, but not in nearby hypothalamic regions (Figure 1E–H). In fact, with the exception of the SCN, co-localization of EYFP and SOX2 was not observed in most brain regions (Figure 1A–D). Our results suggest that SOX2 ablation in the SCN of *Vgat-cre;Sox2^fl/fl^* mice might have occurred on or before E13.5, thus prompting us to profile an earlier developmental age to confirm the time of *Sox2* ablation. To accomplish this, we characterized the expression of EYFP and SOX2 in *Vgat-cre;Sox2^fl/fl^;Rosa^fl/+^* mice, where *Sox2* is conditionally ablated, and *Vgat-cre;Sox2^fl/+^;Rosa^fl/+^* control mice at E12.5 and E13.5 (Figure 2A,B). At both time points, EYFP and SOX2 were detected in the ventral anterior hypothalamus of *Vgat-cre;Sox2^fl/+^;Rosa^fl/+^* control embryos and their expression patterns showed moderate (E12.5) to strong (E13.5) colocalization (Figure 2A,B). In contrast, virtually no EYFP^+^ SOX2^+^ cells were found in the ventral anterior hypothalamus of *Sox2*-deficient embryos, indicating that Cre-mediated excision of *Sox2* took place at or before E12.5 in the *Vgat-cre;Sox2^fl/fl^* model. E12.5 corresponds to the onset of mouse SCN neurogenesis, which peaks at E13.5 and concludes at ~E15 [40]. Notably, VGAT expression is limited to the parenchymal region lateral to the ventricular zone, suggesting that *Sox2* was not ablated from hypothalamic progenitor cells lining the third ventricle (Figure 2A,B). This is consistent with the notion that GABAergic fate determination occurs in the post-mitotic period [41]. Collectively, these data show that in the *Vgat-cre;Sox2^fl/fl^* model, the expression of *Sox2* is ablated specifically in post-mitotic cells of the ventral anterior hypothalamus from E12.5 onwards, while its expression in hypothalamic progenitor cells remains intact throughout embryonic development.

### 2.2. Reduction of SCN Size Is Caused by Postnatal Cell Loss in Sox2-Deficient SCN

**Figure 3:**
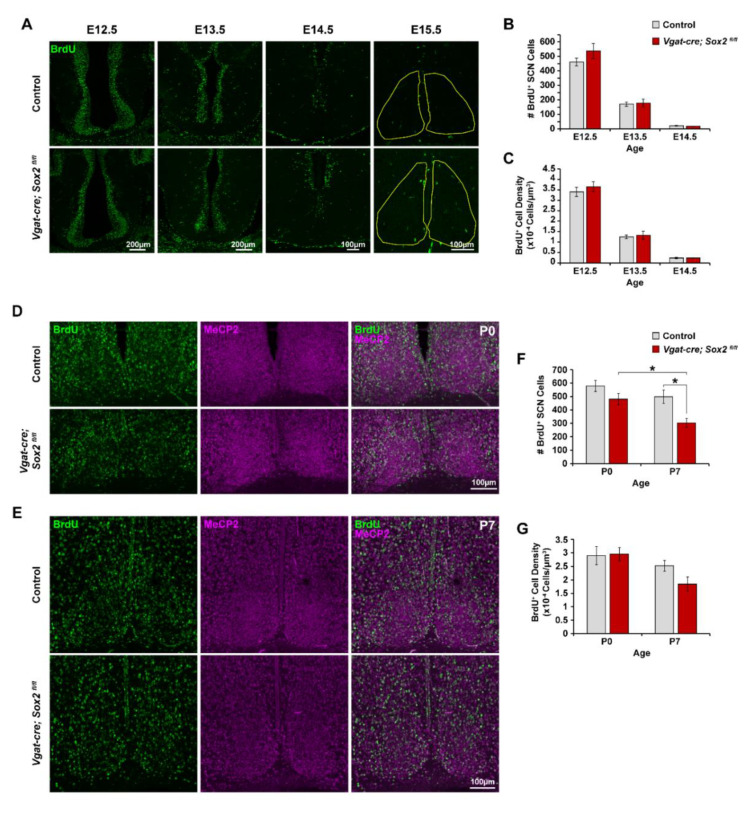
*Vgat-cre;Sox2^fl/fl^* mice exhibit normal cell proliferation during SCN neurogenesis, but fewer newborn neurons survive in the postnatal SCN. (**A**) Representative photomicrographs of BrdU immunoreactivity (green) in the ventral anterior hypothalamus or SCN of control and *Vgat-cre;Sox2^fl/fl^* mice at E12.5, E13.5, E14.5, and E15.5. (**B**) Number of BrdU^+^ cells in the ventral anterior hypothalamus of control and *Sox2* cKO embryos at E12.5, E13.5, and E14.5. (**C**) Quantification of BrdU^+^ cell density in the ventral anterior hypothalamus at E12.5, E13.5, and E14.5. (**D**,**E**) Representative photomicrographs of BrdU (green) and MeCP2 (magenta) immunoreactivity in the (**D**) P0 and (**E**) P7 SCN of control and *Vgat-cre;Sox2^fl/fl^* animals that had received daily pulses of BrdU from E12.5 to E15.5. (**F**,**G**) Quantification of (**F**) number and (**G**) density of BrdU^+^ cells in the P0 and P7 SCN. Values represent mean ± SEM. * *p* < 0.05, two-way ANOVA with Tukey HSD. See Table 1 for the number of animals and litters used.

Given the well-established role of *Sox2* in neurogenesis and the timing of *Sox2* ablation in our mouse model, we asked whether the reduction in SCN cell numbers and size in adult *Sox2* cKO animals may be due to a defective neurogenic program [32,33,34,35]. To examine SCN neurogenesis, we injected timed-pregnant *Sox2^fl/fl^* females that had been mated with *Vgat-cre;Sox2^fl/+^* males with the thymidine analog, bromodeoxyuridine (BrdU), at either E12.5, E13.5, E14.5, or E15.5 to label proliferating cells in the S-phase. Two hours after the injection, embryos were harvested to determine the number and density of BrdU^+^ cells in the SCN region by immunofluorescence labeling. There was no significant difference in the number or density of BrdU^+^ cells between controls (*Sox2^fl/fl^* and *Sox2^fl/+^*) and *Sox2* cKO SCN at E12.5, E13.5, and E14.5, indicating that *Sox2* ablation did not affect cell proliferation during most of the neurogenic period (Figure 3A–C). The number of BrdU^+^ cells at E15.5 was very low in both control and *Sox2* cKO pups, making it difficult to draw conclusions from any quantitative comparison (Figure 3A).

**Table 1:** Animals and litters used for BrdU assays and the corresponding representative figure.

Exp	Age	Litter	*Sox2^fl/fl^* (*n*)	*Sox2^fl/+^*(*n*)	*Sox2* cKO (*n*)	Figure
1	E12.5	A	2	0	1	3A–C
		B	2	0	3	
		Total	4		4	
2	E13.5	A	1	1	3	
		B	2	1	3	
		Total	5		6	
3	E14.5	A	1	1	3	
		B	1	1	1	
		C	0	1	2	
		Total	5		6	
4	P0	A	2	0	2	3D–G
		B	0	0	1	
		C	1	0	1	
		D	1	0	0	
		Total	4		4	
5	P7	A	1	0	2	
		B	0	0	1	
		C	0	0	1	
		D	1	0	1	
		E	2	0	0	
		Total	4		5	

Given the lack of a cell proliferation phenotype, we next examined the impact of *Sox2* ablation on the survival of SCN neurons in the early postnatal stage. Apoptotic cell death in the developing SCN begins as synapse formation increases, with substantial death occurring between P1 and P7 in mice [28,42,43]. To study cell survival, pregnant *Sox2^fl/fl^* dams that had been mated with *Vgat-cre;Sox2^fl/+^* males were given once-daily injections of BrdU during the neurogenic period of E12.5 to E15.5. Pups were collected on P0 and P7 to determine the number and density of BrdU-retaining cells in the SCN before (P0) and after (P7) the peak of SCN cell death. These label-retaining cells, or LRCs, represent the surviving progeny cells of those that were in S-phase at the time of the BrdU injections. There was no significant difference in the number and density of BrdU-retaining cells between P0 *Sox2* cKO pups and littermate controls (*Sox2^fl/fl^* and *Sox2^fl/+^* pups) (Figure 3D,F,G). However, at P7, the number of BrdU^+^ cells in *Sox2* cKO mice was reduced by approximately 24% compared to controls (Figure 3E–G). The density of BrdU-retaining cells at P7 was similar between *Sox2* cKO and control mice, as the mutant SCN was slightly smaller than the control (Figure 3E–G). Taken together, these results suggest that the reduced number of SCN neurons in adult *Sox2* cKO mice is likely stemming from greater neuronal cell loss in the postnatal period rather than a decrease in cell proliferation during SCN neurogenesis.

### 2.3. Loss of Sox2 Leads to Reduced Expression of Neuropeptides

**Figure 4:**
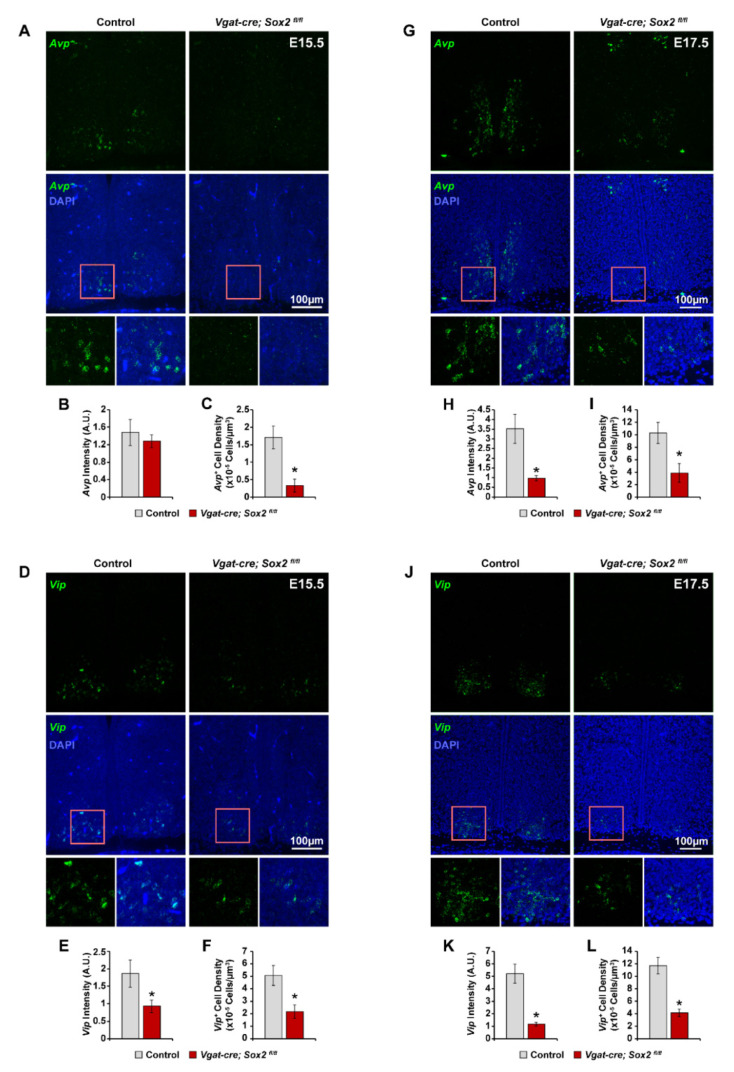
SCN-specific *Sox2* ablation suppresses *Avp* and *Vip* expression at E15.5 and E17.5. (**A**,**G**) Representative photomicrographs of *Avp* expression (green) in the SCN of (**A**) E15.5 and (**G**) E17.5 control and *Vgat-cre;Sox2^fl/fl^* mutant mice. Blue denotes DAPI. (**B**,**H**) Quantification of *Avp* expression in arbitrary units of mean grayscale intensity in the whole SCN at (**B**) E15.5 and (**H**) E17.5. (**C**,**I**) Quantification of *Avp^+^* cell density in the (**C**) E15.5 and (**I**) E17.5 SCN. (**D**,**J**) Representative photomicrographs of *Vip* expression (green) in the SCN of (**D**) E15.5 and (**J**) E17.5 control and *Vgat-cre;Sox2^fl/fl^* mutant mice. Blue denotes DAPI. (**E, K**) Quantification of *Vip* expression in arbitrary units of mean grayscale intensity in the whole SCN at (**E**) E15.5 and (**K**) E17.5. (**F**,**L**) Quantification of *Vip^+^* cell density in the (**F**) E15.5 and (**L**) E17.5 SCN. Values represent mean ± SEM. * *p* < 0.05 versus control mice, two-tailed Student’s *t*-test. See Table 2 for the number of animals and litters used.

**Figure 5:**
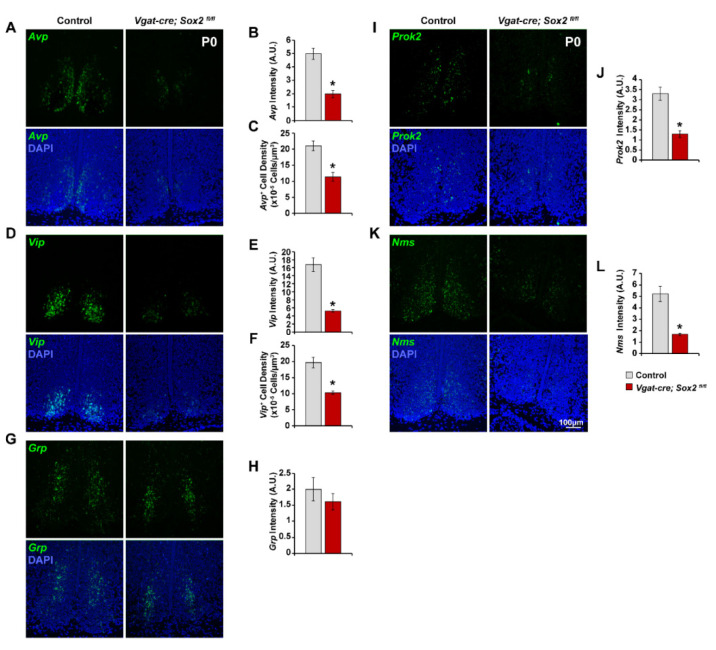
Effects of *Sox2* ablation on neuropeptide expression in P0 SCN. (**A**,**D**,**G**,**I**,**K**) Representative photomicrographs of (**A**) *Avp*, (**D**) *Vip*, (**G**) *Grp*, (**I**) *Prok2*, and (**K**) *Nms* expression (green) in the SCN of P0 control and *Vgat-cre;Sox2^fl/fl^* mutant mice. Blue denotes DAPI. (**B**,**E**,**H**,**J**,**L**) Expression of (**B**) *Avp*, (**E**) *Vip*, (**H**) *Grp*, (**J**) *Prok2*, and (**L**) *Nms* at P0 are quantified in arbitrary units of mean grayscale intensity in the whole SCN. (**C**,**F**) Quantification of (**C**) *Avp^+^* and (**F**) *Vip^+^* cell density in the P0 SCN. Values represent mean ± SEM. * *p* < 0.05 versus control mice, two-tailed Student’s *t*-test. See Table 2 for the number of animals and litters used.

Next, we examined when *Sox2*-deficient SCN neurons acquire the expression of signature neuropeptides as an indicator of their developmental progression. We profiled the expression pattern of *Avp* and *Vip* in E15.5 and E17.5 SCN with fluorescence in situ hybridization. Expression of *Avp* and *Vip* was low but detectable in control (*Sox2^fl/fl^* and *Sox2^fl/+^*) SCN at E15.5 (Figure 4A,D). *Avp^+^* cells were concentrated in the medial region proximal to the third ventricle (Figure 4A), while the majority of *Vip^+^* cells were situated in the lateral SCN (Figure 4D). There was no significant difference in *Avp* expression intensity between E15.5 *Sox2* cKO and control SCN (Figure 4B). However, the density of *Avp^+^* cells was decreased by ~80% in *Sox2*-deficient SCN at E15.5 compared to control SCN (Figure 4A,C). *Sox2* ablation also reduced the expression of *Vip* and the density of *Vip^+^* cells in E15.5 SCN by 50% and 57%, respectively (Figure 4D–F). Between E15.5 and E17.5, the density of *Avp^+^* and *Vip^+^* cells in control SCN increased by 6.25- and 2.4-fold, respectively, as more SCN neurons differentiated and began to express neuropeptides (Figure 4C,F,I,L). A similar phenomenon was observed in *Sox2*-deficient SCN in the same period, during which the density of *Avp^+^* cells and *Vip^+^* cells increased by 10- and 2-fold, respectively (Figure 4C,F,I,L). However, at E17.5, *Sox2*-deficient SCN continued to exhibit significantly lower expression of *Avp* and *Vip* and lower density of *Avp^+^* and *Vip^+^* cells relative to control SCN (Figure 4C,F,I,L). From E17.5 to P0, the density of *Avp^+^* cells in control and *Sox2* cKO SCN further increased by 2- and 2.5-fold, respectively (Figure 4I and Figure 5C). *Avp^+^* cell density was reduced by ~45% in P0 *Sox2*-deficient SCN relative to controls, with the remaining *Avp^+^* cells localized to the medial SCN and absent from the lateral SCN (Figure 5A,C). Between E17.5 and P0, there was also an increase of 1.7- and 2.4-fold in the density of *Vip^+^* cells in control and *Sox2*-deficient SCN, respectively (Figure 4L and Figure 5F). *Vip*^+^ cell density was reduced by ~47% in P0 *Sox2* cKO animals relative to controls, with the compartmentalization of *Vip^+^* cells largely resembling that of wild-type animals (Figure 5D,F). In addition to *Avp* and *Vip*, we characterized the expression profile of three other SCN neuropeptides, *Grp*, *Nms*, and *Prok2*, in P0 control and *Sox2*-deficient SCN. The levels of *Nms* and *Prok2*, but not *Grp*, were significantly reduced in *Sox2* cKO SCN compared to controls (Figure 5G–L). Collectively, our findings suggest that the absence of SOX2 in the developing SCN interferes with the ability of SCN neurons to acquire the expression of their signature neuropeptides.

**Table 2:** Animals, litters, RNAscope probes used and the corresponding representative figures.

Exp.	Age	RNAscope Probe (Identifier)	Figure	*Sox2^fl/fl^*	*Sox2^fl/+^*	*Sox2* cKO
				(Litter—*n*)		
1	E12.5	*Lhx1* (488581)	6A	A—2; B—2	A—0; B—0	A—1; B—3
		*Rax* (528561-C2)	6A			
2	E14.5	*Six6* (574291)	6F	A—1; B—1; C—0	A—1; B—1; C—1	A—3; B—1; C—2
		*Lhx1* (488581-C2)	6B			
		*Six3* (412941-C3)	6D			
3	E15.5	*Lhx1* (488581)	6H	A—1; B—1; C—1; D—1	A—0; B—0; C—0; D—0	A—1; B—1; C—1; D—1
		*Avp* (401391-C2)	4A			
		*Vip* (415961-C3)	4D			
4	E15.5	*Six6* (574291)	6L	A—3; B—1; C—1	A—0; B—0; C—0	A—2; B—1; C—1
		*Six3* (412941-C3)	6J			
5	E17.5	*Six6* (574291)	6P	A—1; B—3	A—1; B—0	A—2; B—2
		*Lhx1* (488581-C2)	6N			
		*Avp* (401391-C3)	4G*			
6	E17.5	*Vip* (415961-C3)	4J			
7	P0	*Grp* (317861)	5G	A—3; B—1	A—0; B—0	A—3; B—1
12	P0	*Six6* (574291)	7E			
		*Vax1* (805101-C2)	7K			
8	P0	*Prok2* (447941)	5I	A—1; B—1; C—2	A—0; B—1; C—0	A—1; B—2; C—3
9	P0	*Nms* (472331)	5K			
10	P0	*Lhx1* (488581)	7A	A—1; B—1; C—1; D—1	A—0; B—0; C—0; D—0	A—1; B—1; C—1; D—1
		*Avp* (401391-C2)	5A			
		*Vip* (415961-C3)	5D			
11	P0	*Rora* (520031)	7G	A—1; B—0; C—2	A—0; B—0; C—0	A—1; B—1; C—1
		*Rorb* (444271-C2)	7I			
		*Six3* (412941-C3)	7C			

* A different control is shown as the representative photomicrograph.

### 2.4. Ablation of SOX2 in SCN Neurons Impairs Lhx1 and Six6 Expression at Birth but Not during Embryonic Development

**Figure 6:**
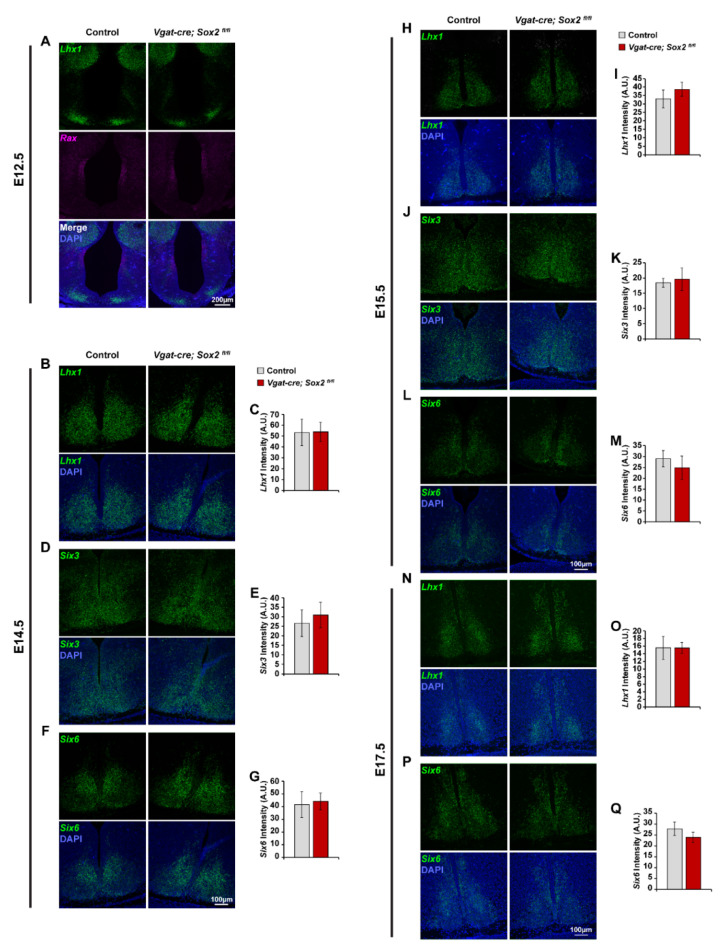
The expression of major SCN-enriched transcription factors is preserved in the embryonic, *Sox2*-deficient SCN. (**A**) Representative photomicrographs of *Lhx1* (green) and *Rax* (magenta) expression in the anterior hypothalamus of E12.5 control and *Vgat-cre;Sox2^fl/fl^* mutant embryos. Blue denotes DAPI. (**B**,**D**,**F**) Representative photomicrographs of (**B**) *Lhx1*, (**D**) *Six3*, and (**F**) *Six6* expression (green) in the E14.5 SCN of control and *Vgat-cre;Sox2^fl/fl^* mutant embryos. Blue denotes DAPI. (**C**,**E**,**G**) The expression of (**C**) *Lhx1*, (**E**) *Six3*, and (**G**) *Six6* at E14.5 are quantified in arbitrary units of mean grayscale intensity in the whole SCN. (**H**,**J**,**L**) Representative photomicrographs of (**H**) *Lhx1*, (**J**) *Six3*, and (**L**) *Six6* expression (green) in the E15.5 SCN of control and *Vgat-cre;Sox2^fl/fl^* mutant embryos. Blue denotes DAPI. (**I**,**K**,**M**) The expression of (**I**) *Lhx1*, (**K**) *Six3*, and (**M**) *Six6* at E15.5 are quantified in arbitrary units of mean grayscale intensity in the whole SCN. (**N, P**) Representative photomicrographs of (**N**) *Lhx1* and (**P**) *Six6* expression (green) in the E17.5 SCN of control and *Vgat-cre;Sox2^fl/fl^* mutant embryos. Blue denotes DAPI. (**O, Q**) The expression of (**O**) *Lhx1* and (**Q**) *Six6* at E17.5 are quantified in arbitrary units of mean grayscale intensity in the whole SCN. Values represent mean ± SEM. * *p* < 0.05 versus control mice, two-tailed Student’s *t*-test. See Table 2 for the number of animals and litters used.

**Figure 7:**
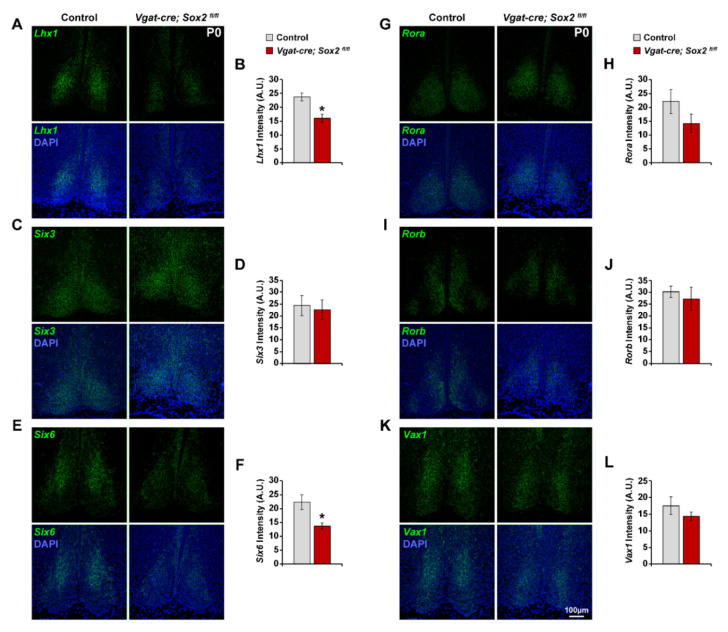
Loss of SOX2 disrupts *Lhx1* and *Six6* expression in P0 *Vgat-cre;Sox2^fl/fl^* SCN. (**A**,**C**,**E**,**G**,**I**,**K**) Representative photomicrographs of (**A**) *Lhx1*, (**C**) *Six3*, (**E**) *Six6*, (**G**) *Rora*, (**I**) *Rorb*, and (**K**) *Vax1* expression (green) in the SCN of P0 control and *Vgat-cre;Sox2^fl/fl^* mutant mice. Blue denotes DAPI. (**B**,**D**,**F**,**H**,**J**,**L**) The expression of (**B**) *Lhx1*, (**D**) *Six3*, (**F**) *Six6*, (**H**) *Rora*, (**J**) *Rorb*, and (**L**) *Vax1* at P0 are quantified in arbitrary units of mean grayscale intensity in the whole SCN. Values represent mean ± SEM. * *p* < 0.05 versus control mice, two-tailed Student’s *t*-test. See Table 2 for the number of animals and litters used.

The SCN expresses many transcription factors at different developmental stages, and a small subset of these genetic markers has been confirmed to regulate SCN specification and terminal differentiation [23,25,28]. To address the possibility that *Sox2* may be modulating SCN development by controlling the expression of known transcription factors, we characterized the expression of three major SCN specification and differentiation factors, *Lhx1*, *Six3*, and *Six6*, in the developing SCN of *Sox2* cKO and control (*Sox2^fl/fl^* and *Sox2^fl/+^*) mice. There was no significant difference in the expression of the SCN differentiation factor, *Lhx1*, at E14.5, E15.5, and E17.5 (Figure 6B,C,H,I,N,O). The level and distribution of *Lhx1* expression appeared to be similar between *Sox2* cKO and control SCN at E12.5, which was also the case for *Rax*, a transcription factor that is expressed in the early neuroepithelium (Figure 6A). The expression of the SCN specification factor, *Six6*, in *Sox2*-deficient SCN was intact at E14.5, E15.5, and E17.5, consistent with the preservation of a histologically recognizable SCN in our mutant mice (Figure 6F,G,L,M,P,Q; [23]). *Sox2* ablation did not affect the expression of another early specification marker of the SCN, *Six3,* at either E14.5 or E15.5 (Figure 6D,G,J,K; [25]). However, expression of *Lhx1* and *Six6*, but not *Six3*, was eventually disrupted by *Sox2* ablation at P0: *Lhx1* and *Six6* were significantly attenuated in the dorsomedial SCN of *Sox2* cKO mice, mirroring the phenotype seen in the adults (Figure 7A,B,E,F) [37,38].

To confirm that the downregulation of *Lhx1*, *Six6*, and various neuropeptides in the SCN was not the result of a failure in SCN compartmentalization, we examined the expression of the dorsomedial SCN-specific marker, *Rorb*, in P0 animals. *Rorb* retained its normal compartmentalization and intensity when *Sox2* was ablated (Figure 7I,J). We also assessed the expression of two other selective SCN markers, *Rora* and *Vax1*. *Rora* and *Vax1* exhibited a largely normal expression profile with no significant difference in signal intensity between *Sox2*-deficient and control SCN (Figure 7G,H,K,L). In summary, the data suggest that *Sox2* deficiency in the SCN downregulates the expression of *Lhx1* and *Six6* at P0, but not during embryogenesis when neuronal specification normally takes place.

## 3. Discussion

Our initial characterization of *Vgat-cre;Sox2^fl/fl^* mice revealed imprecise, poorly consolidated behavioral rhythms that entrained inefficiently to light, but also a visibly smaller SCN compared to control mice [37]. The latter observation raised the possibility that *Vgat*-driven *Sox2* ablation may negatively influence the development of the SCN. Here, we report that SOX2 and VGAT expression colocalize in the developing SCN at E12.5, and that Cre-mediated gene excision begins during early SCN neurogenesis. Despite the early excision of *Sox2* from GABAergic cells, *Vgat-cre;Sox2^fl/fl^* mice exhibit normal SCN neurogenesis comparable to littermate controls. The reduction in SCN size is the consequence of enhanced cell loss in the postnatal SCN of *Sox2*-deficient mice rather than a decrease in cell proliferation during embryogenesis. In the absence of SOX2, the expression of several SCN-enriched neuropeptides including *Avp*, *Vip*, *Prok2*, and *Nms* is markedly diminished, some as early as E15.5. This suggests that *Sox2* ablation may impede the differentiation of neuropeptidergic neurons, potentially contributing to the circadian phenotype of adult *Vgat-cre;Sox2^fl/fl^* mice.

Using a Cre-reporter mouse strain, we confirmed that SCN-specific *Sox2* ablation in the *Vgat-cre;Sox2^flfl^* mouse model occurs on or before E12.5. Notably, SOX2 is concentrated at the neuroepithelium lining the third ventricle, while EYFP, a proxy for Cre and *Vgat* expression, has moderate colocalization with SOX2 in the ventral anterior hypothalamus beginning at E12.5. More SOX2^+^ EYFP^+^ cells are present in the parenchymal region lateral to the third ventricle at E13.5, and *Vgat*-driven expression of Cre essentially ablates SOX2 from this population of cells, which will eventually form the SCN. Neuroepithelial expression of SOX2 is intact throughout development as *Vgat* is not expressed in this region. At later timepoints, colocalized expression of SOX2 and EYFP remains confined to the SCN and is virtually undetected in other brain regions including other hypothalamic nuclei.

SOX2 is a neuronal stem/progenitor cell marker that labels mitotic cells in neurogenic niches during embryonic and adult neurogenesis. On the other hand, *Vgat* is considered to be a marker of terminally differentiated cells: it is expressed in postmitotic, GABAergic neurons and confers cell type-specific functional properties [41]. Using *Vgat* to drive *Sox2* excision essentially protects proliferating progenitors as well as early postmitotic, non-GABAergic precursors from *Sox2* deletion. The mutually exclusive nature of *Vgat* and *Sox2* expression in major neurogenic niches also allows for normal genesis of the nervous system in *Vgat*-driven *Sox2* cKO animals and prevents the major deficiencies observed in constitutive, *Foxg1*- or *Nestin*-driven *Sox2* KO mouse models [32,33,34,35,36]. Furthermore, *Sox2* is usually suppressed when proliferating progenitors are triggered to exit the cell cycle by proneural genes. However, this does not apply to SCN neurons, as their expression of *Sox2* is retained throughout differentiation and maturation, rendering them susceptible to the effects of *Vgat*-driven *Sox2* deletion. Indeed, the *Vgat-cre;Sox2^fl/fl^* model provides a unique intersectional approach to delineate the function of *Sox2* in SCN development independent of its role in pluripotency or the proliferation of most neural progenitors.

By labeling S-phase cells with a thymidine analog, we found that cell proliferation during the window of SCN neurogenesis is not affected by *Sox2* deletion. These data suggest that the reduced SCN volume observed in adult *Sox2* cKO mice is unlikely to be the consequence of a proliferation defect in SCN neuronal precursors. They are also consistent with the observation that *Vgat*-driven *Sox2* ablation does not perturb the expression of SOX2 in proliferating hypothalamic progenitors lining the third ventricle as recombination occurs only in post-mitotic cells. In contrast, the BrdU retention experiment revealed that significantly fewer of these previously dividing cells survive to P7 in the absence of SOX2, accounting for the reduction in SCN volume.

Cheng et al. (2019a) established that *Sox2* deletion dramatically downregulates the expression of neuropeptides, neuropeptide receptors, and core clock genes in adult mice, causing disrupted circadian clock function [37]. Transcription factors with critical developmental roles, such as *Lhx1* and *Six6*, are also differentially expressed in *Sox2*-deficient, adult SCN. This prompted us to consider potential defects in terminal differentiation or early SCN specification, akin to the phenotypes elicited by ventral anterior hypothalamus-specific deletion of *Lhx1* and constitutive deletion of *Six6*, respectively [23,28]. Our ISH results showed that the SCN of *Sox2*-deficient P0 mice already exhibit drastically lower expression of *Avp*, *Vip*, *Lhx1*, and *Six6*, suggesting that *Sox2* ablation is detrimental to SCN development. This observation gave rise to three non-mutually exclusive hypotheses that could explain the developmental defects in *Sox2* cKO mice. First, SOX2 might regulate *Six6* expression: *Sox2* ablation would disrupt the *Six6* transcriptional program leading to a partial failure of SCN specification and patterning, and ultimately the loss of *Lhx1*-mediated SCN terminal differentiation. Second, *Sox2* might regulate *Lhx1* expression independently of *Six6*: deleting *Sox2* would lead to a downregulation of *Lhx1* and the subsequent loss of neuropeptidergic neuronal populations. In support of these hypotheses, in silico analyses reported in Cheng et al. (2019b) suggested that SOX2 might directly regulate *Six6* and *Lhx1* expression in the SCN [38]. Third, differentiation and maturation of SCN neurons could be regulated by *Sox2* either directly or through uncharacterized pathways that operate independently of *Lhx1* and *Six6*. To determine the validity of these hypotheses, we examined the expression of *Avp*, *Vip*, *Lhx1,* and *Six6* in the SCN at E14.5, E15.5, and E17.5. We found that the numbers of *Avp^+^* and *Vip^+^* cells are drastically reduced in the SCN of E15.5 and E17.5 *Sox2* cKO mice, while the expression of *Lhx1* and *Six6* is unaffected at all embryonic time points. The results suggest that deficits in differentiation of *Avp^+^* and *Vip^+^* neurons arising from *Sox2* ablation precede, and thus do not require, the downregulation of *Lhx1* and *Six6*, consistent with our third hypothesis. It is plausible that reduced expression of *Lhx1* and *Six6* at later developmental stages further exacerbates the differentiation phenotype or affects neuronal maturation, but this remains a provisional explanation. Moreover, it is unclear whether SOX2 can directly regulate the transcription of SCN-enriched neuropeptides given the lack of consensus SOX2 binding sites in their gene promoters or enhancer regions [44]. However, more empirical data is required to rule out this possibility.

It is worth noting that cell attrition is not the primary cause for neuropeptide and transcription factor downregulation, as many of these genes already exhibit reduced expression in *Sox2*-deficient SCN during gestation (*Avp* and *Vip*) or at P0 (*Prok2*, *Nms, Lhx1, and Six6*), prior to significant neuronal loss. Rather, our data suggest that increased cell death is likely secondary to defects in SCN terminal differentiation in *Sox2 cKO* mice, where incompletely differentiated or functionally impaired neurons are cleared by programmed cell death. In support of our hypothesis, direct neuronal reprogramming studies have shown that cells which fail to convert successfully to neurons will instead succumb to cell death [45]. A more detailed characterization of SCN cell death in *Sox2*-deficient mice should be conducted to determine the underlying mechanism.

Discrepancies with the published literature on the onset and distribution of key clock components are noted. Onsets of *Avp* and *Vip* expression have been described to take place at the late embryonic stage (~E18) in mice. However, we were able to detect clear mRNA signals of both neuropeptides as early as E15.5, which is 2.5 days earlier than previously reported in mice and other rodents [25,46,47,48]. One possible explanation for our earlier detection may be the greater sensitivity of the RNAscope technology compared to traditional ISH methods [49,50]. An alternative, but less likely, explanation is that animals with a *Sox2^fl/fl^* background have an earlier neuropeptide expression. In terms of *Lhx1* expression, VanDunk et al. (2011) described a dynamic expression pattern throughout the development of the SCN [25]. In that study, *Lhx1* is first detected in the anterior hypothalamus at E11.5, gradually spans the entire SCN by E15.5, and then appears to be confined to the medial SCN from E17.5 onwards [25]. In contrast, Bedont et al. (2014) reported that *Lhx1* expression spans the entire SCN throughout development and after birth [28]. Our results agree with both studies with respect to the onset of *Lhx1* expression, but align specifically with the findings of Bedont et al. (2014) in terms of the broad distribution of *Lhx1* throughout the SCN from E14.5 to P0 [28].

Our study reveals that the ablation of *Sox2* during early SCN development disrupts *Avp* and *Vip* expression in the embryonic SCN prior to downregulation of *Lhx1* and *Six6*. The ablation of *Sox2* likely disrupts differentiation and maturation of neuropeptidergic neurons in the SCN in a *Six6*- and *Lhx1*-independent manner, ultimately compromising their ability to survive during the postnatal cell clearance window. These results suggest that some of the phenotypes observed in adult *Sox2* cKO mice may stem from developmental deficits [37,51]. Downregulated neuropeptide expression in the adult mutant SCN could be explained by immature SCN neurons that fail to differentiate. Although we have only examined the developmental trajectory of the two major populations of peptidergic neurons in *Sox2*-deficient SCN, other populations including *Nms^+^* and *Prok2^+^* neurons may have similar impairments in differentiation that account for the diminished expression of their neuropeptides in the adult SCN. This does not preclude the possibility that SOX2 may regulate the transcription of these neuropeptide-encoding genes, either directly or indirectly, beyond the developmental stage. Given the importance of neuropeptides in the communication between SCN neurons and to efferent targets, the behavioral phenotypes of *Sox2* cKO mice that are associated with disrupted intercellular signaling may be partially attributed to abnormal SCN differentiation [37]. For example, a defect in the differentiation of peptidergic populations within the SCN that signal to other brain regions regulating affective behavior may underlie the anxio-depressive-like phenotypes exhibited by *Sox2* cKO mice [51]. In addition to reduced neuropeptide expression, enhanced cell loss results in a smaller SCN network, which would likely impact clock network dynamics and stability in *Sox2*-deficient SCN; however, further experiments are needed to establish the specific effects of a smaller oscillator. It is also not clear whether altered intra-SCN communication is responsible for the blunted expression of several core clock genes, including *Arntl*, *Cry1*, *Per2*, and *Rora,* in the SCN of adult *Sox2* cKO mice. We do not believe this to be the case, as these clock genes are downregulated at only one (or, in the case of *Arntl*, two) time point(s), and the magnitude of their downregulation is less striking compared to that of differentially expressed neuropeptides and their receptors [37,38]. Overall, our study positions *Sox2* as a novel differentiation factor within the SCN that is required for the development of major peptidergic populations.

## 4. Materials and Methods

### 4.1. Animals

All animal handling and experimental procedures were performed at the University of Toronto Mississauga (UTM) Animal Facility and were approved by the UTM Animal Care Committee, complying with guidelines established by the University of Toronto Animal Care Committee and the Canadian Council on Animal Care. The following mouse strains were purchased from The Jackson Laboratory (Bar Harbor, ME, USA) and bred in-house to generate the appropriate genotypes for this study: homozygous *Sox2^fl/fl^* mice in which the coding exon of *Sox2* is flanked by loxP sequences (*Sox2^tm1.1Lan^*); homozygous *Vgat*-IRES-Cre (*Vgat^cre/cre^*) knock-in mice in which the IRES-Cre recombinase cassette is inserted downstream of the stop codon of the endogenous vesicular GABA transporter (*Vgat*) gene (*Slc32a1^tm2(cre)Lowl^*); and homozygous *Rosa26-loxP-STOP-loxP-EYFP* (*Rosa^fl/fl^*) mice in which EYFP expression is induced by Cre/loxP-mediated excision of an upstream STOP sequence (*Gt(ROSA)26Sor^tm1(EYFP)Cos^*). *Vgat^cre/cre^* mice were bred to *Sox2^fl/fl^* mice, and a breeding colony was maintained by mating *Sox2^fl/fl^* mice with *Vgat^cre/+^;Sox2^fl/+^* mice. *Vgat^cre/cre^* mice were mated with *Rosa^fl/fl^* animals to generate *Vgat^cre/+^;Rosa^fl/+^* mice for mapping of VGAT and SOX2 expression. To confirm loss of SOX2 expression, *Sox2^fl/fl^;Rosa^fl/fl^* mice were bred with *Vgat^cre/+^;Sox2^fl/+^* mice to obtain *Vgat^cre/+^;Sox2^fl/fl^;Rosa^fl/+^* (*Sox2* conditional knockout) and *Vgat^cre/+^;Sox2^fl/+^;Rosa^fl/+^* (*Sox2* heterozygous) animals for analysis. Littermate *Sox2^fl/fl^* and *Sox2^fl/+^* mice were used as controls for all other experiments. Mice were bred and maintained on a fixed 12-hr light:12-hr dark (12:12 LD) schedule in which lights on and lights off corresponded to 8 am and 8 pm Eastern Standard Time, respectively. Females were inspected for the presence of a vaginal plug each morning (2–3 h after lights on) and were housed separately from the male breeder as soon as a vaginal plug was detected.

### 4.2. Tissue Harvest

For the staging of embryos, the morning of the vaginal plug formation was designated as E0.5. For staging of neonates, the date of birth was considered P0. All tissue harvests were performed between zeitgeber time (ZT) 11 and 12, at the end of the light phase. Timed pregnant females were killed by cervical dislocation and their embryos were quickly extracted and placed in ice-cold, RNase-free PBS. Embryos were decapitated and their heads were fixed in RNase-free 4% paraformaldehyde (PFA) in PBS overnight at 4 °C, washed in diethyl pyrocarbonate (DEPC)-treated PBS, and cryoprotected in 30% sucrose in PBS for at least 24 h. Tissues were then frozen in Tissue Tek Optimal Cutting Temperature (OCT) embedding media (Sakura Finetek, Torrance, CA, USA) with dry ice and cut into 14 µm thin sections using the CryoStar NX50 Cryostat (Thermo Scientific, Waltham, MA, USA). Sections were thaw mounted on SuperFrost Plus slides (Fisher Scientific, Waltham, MA, USA). Slides were left in −20 °C for 2 h, and then stored at −80 °C until further processing.

To examine bromodeoxyuridine (BrdU) retention and EYFP expression in P0 mice, animals were decapitated, and their brains were rapidly dissected and fixed in 4% PFA in PBS overnight at 4 °C. Afterwards, they were washed in PBS and cryoprotected in 30% sucrose in PBS for at least 24 h. Tissues were cut into 30 μm or 40 μm thin sections using a freezing microtome (Leica Microsystems, Wetzlar, Germany), and stored in 30% sucrose at 4 °C until further use. For in situ hybridization, P0 mice were decapitated, and their brains were fixed in RNase-free 4% PFA in PBS overnight at 4 °C, washed in DEPC-treated PBS, and cryoprotected in 30% sucrose in PBS for at least 24 h. Tissues were processed and stored as described above for embryonic brain tissues.

P7 and adult brains were dissected and sectioned in ice-cold oxygenated media with an oscillating tissue slicer (Electron Microscopy Sciences, Hatfield, PA, USA) to obtain 800 μm thick coronal slices. Tissue slices were fixed in 4% PFA in PBS (pH 7.4) for 6 h at room temperature, cryoprotected in 30% sucrose in PBS at 4 °C overnight, cut into 30 μm (adult) and 40 μm (P7) thin sections using a freezing microtome (Leica Microsystems), and stored in 30% sucrose at 4 °C until further use.

### 4.3. Immunofluorescence (IF)

Tissues were washed 5 × 5 min in PBS (pH 7.4) with 0.1% Triton X-100 (PBST), incubated for 1 h at room temperature (RT) in blocking solution (10% heat inactivated horse serum (Wisent Inc., Saint-Jean-Baptiste, QC, Canada) in PBST), and incubated overnight at 4 °C in fresh blocking solution containing the primary antibody (Goat anti-GFP, 1:1000, Eusera, Edmonton, AB, Canada, EU3; Rabbit anti-SOX2, 1:300, Abcam, Cambridge, UK, ab97959). The next day, tissues were washed 5 × 5 min with PBST and incubated for 2 h at RT, protected from light, with the appropriate secondary AlexaFluor antibody (1:1000, ThermoFisher Scientific, Waltham, MA, USA) diluted in blocking solution. Sections were washed 5 × 5 min with PBST, incubated for 10 min in DAPI (1 μg/mL, Sigma-Aldrich, St. Louis, MO, USA) diluted in PBS, washed twice in PBS, and mounted onto glass microscope slides. Slides were coverslipped with Vectashield Mounting Medium (Vector Biolabs, Malvern, PA, USA) and sealed with nail polish.

### 4.4. Bromodeoxyuridine Labeling

Pregnant female mice were injected intraperitoneally with BrdU (100 mg/kg, Sigma-Aldrich, B5002) at the specified embryonic age between ZT9 and ZT10. Embryos and pups were fixed and sectioned as described above. Tissues were treated with 2N HCl for 25 min at 55 °C, washed 3 times in PBS at room temperature, and incubated in 10% heat inactivated horse serum (Wisent Inc.) in PBST for 1 h. The slides were incubated overnight at 4 °C in fresh blocking solution containing rat anti-BrdU antibody (1:1000, Bio-Rad Laboratories, Hercules, CA, USA, OBT0030G) and rabbit anti-MeCP2 antibody (1:200, EMD Millipore, Burlington, MA, USA, 07-013). The next day, tissues were washed 5 × 5 min with PBST and incubated for 2 h at RT in the dark with the appropriate secondary AlexaFluor antibody (1:1000, ThermoFisher Scientific) diluted in blocking solution. Sections were washed 5 × 5 min with PBST, followed by one PBS rinse. Free-floating sections from P0 and P7 animals were mounted onto glass microscope slides. Slides were coverslipped with Vectashield Mounting Medium (Vector Biolabs) and sealed with nail polish.

### 4.5. RNAscope in situ Hybridization (ISH)

The in situ hybridization (ISH) probes that were used to visualize mRNA expression were purchased from Advanced Cell Diagnostics (Newark, CA, USA). The probes used in this study can be found in Table 2. The RNAscope Multiplex Fluorescent Reagent Kit v2 (Cat #323100, Advanced Cell Diagnostics, Newark, CA, USA) was used according to the manufacturer’s instructions for fixed frozen sections with some modifications. Slides were washed in PBS for 5 min, baked at 60 °C for 30 min, fixed with 4% PFA in PBS for 90 min at RT, dehydrated in increasing concentrations of ethanol (50%, 70%, 100%, and 100%), and baked again at 60 °C for 10 min. Slides were then treated with RNAscope hydrogen peroxide for 10 min at RT. Antigen retrieval was performed by placing slides into mildly boiling RNAscope target retrieval reagent for 5 min and then washing the slides in distilled water for 15 s, followed by 100% ethanol for 3 min. Slides were dried and permeabilized with RNAscope Protease III for 30 min at 40 °C. Next, slides were rinsed with distilled water followed by probe incubation for 2 h at 40 °C. Afterwards, slides were incubated with a series of amplifier probes and HRP solution at 40 °C (AMP1, 30 min; AMP2, 30 min; AMP3, 15 min; HRP-C1, HRP-C2 or HRP-C3, 15 min) with 2 × 2 min of washes with RNAscope wash buffer in between each step. Slides were incubated with Opal dyes (Akoya Biosciences, Menlo Park, CA, USA) diluted in TSA buffer for 30 min at 40 °C. HRP activity was then blocked, sections were counterstained with DAPI, and slides were coverslipped with Vectashield Mounting Medium (Vector Biolabs). Positive and negative control probes (identifier: 320881 and 320871) were used in all experiments to ensure RNA quality and probe specificity.

### 4.6. Image Acquisition and Quantification

Images were acquired using a Zeiss Axio Observer Z1 inverted microscope equipped with a Laser Scanning Microscope (LSM) 700 module for confocal images and an AxioCam MRm Rev.3 monochromatic digital camera (Zeiss, Jena, Germany) for bright-field pictures, with the Zen 2010 software (Zeiss). Identical settings were used for imaging samples within each experiment (gain, pinhole, filter sets for confocal microscopy). Confocal images were acquired in separate channels for each fluorophore. All image analyses were performed on ImageJ.

*Intensity.* For quantification of staining intensity, 20× confocal images of the bilateral SCN were acquired. The area of each SCN was delineated using the polygon selection tool based on the higher cell density as reflected by the DAPI staining pattern, and the average grayscale intensity was obtained using the “measure” function. Background staining was measured in a non-immunoreactive region adjacent to each SCN and subtracted from the immunoreactive intensity within the SCN.

*Cell counts.* For BrdU^+^ cell count and density measurement, 20× confocal images were acquired that covered the entire surface of the bilateral SCN. Thirty- or 40-μm-thick coronal sections were used for P0 and P7 mice. The SCN area for P0 and P7 mice was measured and delineated using the polygon tool based on the higher cell density as reflected by MeCP2 immunoreactivity. For E12.5, E13.5, E14.5, and E15.5 SCN, tissues were serially sectioned and every 14-μm-thick coronal section was mounted onto pairs of microscope slides in an alternating fashion. One series of tissues (corresponding to the 1st, 3rd, 5th, etc., sections) was labelled with anti-BrdU antibody, and the other series (corresponding to the 2nd, 4th, 6th, etc., sections) was labelled with *Lhx1* RNAscope probe. *Lhx1* expression pattern was used to define the boundary of the SCN, which was then inferred to the neighboring/adjacent sections used for BrdU IHC by common hypothalamic landmarks. The total number of BrdU^+^ cells within the SCN of 3 to 5 central coronal sections was counted manually through all z-planes by an observer blind to the genotype of the mice. The average number and density of BrdU^+^ cells in one unilateral SCN slice from each animal was calculated. The values reported represent mean ± SEM of 3 to 5 animals of the same experimental group (see Table 1).

For quantification of *Avp^+^* and *Vip^+^* cells, 20× confocal images were acquired that covered the entire surface of the bilateral SCN from 14-μm-thick coronal sections (for all age points). The SCN area was first measured and delineated using the polygon tool based on the higher cell density as reflected by the DAPI staining pattern. The total number of *Avp^+^* and *Vip^+^* cells within the SCN of 3 to 5 central coronal sections was counted manually through all z-planes. The average number and density of *Avp^+^* and *Vip^+^* cells in one unilateral SCN slice from each animal was calculated. The values reported represent mean ± SEM of 3 to 5 animals of the same experimental group (see Table 2).

### 4.7. Statistical Analysis

Data were analyzed using two-tailed Student’s *t*-test and two-way ANOVA with R version 4.0.3. *Post hoc* significance of pairwise comparisons was assessed using Tukey’s Honest Significant Difference test with α set at 0.05.

## Data Availability

Data supporting reported results may be supplied upon request by authors.

## References

[1] Inouye S.T., Kawamura H. (1979). Persistence of circadian rhythmicity in a mammalian hypothalamic “island” containing the suprachiasmatic nucleus. Proc. Natl. Acad. Sci. USA.

[2] Welsh D.K., Logothetis D.E., Meister M., Reppert S.M. (1995). Individual neurons dissociated from rat suprachiasmatic nucleus express independently phased circadian firing rhythms. Neuron.

[3] Gekakis N., Staknis D., Nguyen H.B., Davis F.C., Wilsbacher L.D., King D.P., Takahashi J.S., Weitz C.J. (1998). Role of the CLOCK protein in the mammalian circadian mechanism. Science.

[4] Kume K., Zylka M.J., Sriram S., Shearman L.P., Weaver D.R., Jin X., Maywood E.S., Hastings M.H., Reppert S.M. (1999). mCRY1 and mCRY2 are essential components of the negative limb of the circadian clock feedback loop. Cell.

[5] Bunger M.K., Wilsbacher L.D., Moran S.M., Clendenin C., Radcliffe L.A., Hogenesch J.B., Simon M.C., Takahashi J.S., Bradfield C.A. (2000). Mop3 Is an Essential Component of the Master Circadian Pacemaker in Mammals. Cell.

[6] Vitaterna M.H., Ko C.H., Chang A.M., Buhr E.D., Fruechte E.M., Schook A., Antoch M.P., Turek F.W., Takahashi J.S. (2006). The mouse Clock mutation reduces circadian pacemaker amplitude and enhances efficacy of resetting stimuli and phase-response curve amplitude. Proc. Natl. Acad. Sci. USA.

[7] Shearman L.P., Zylka M.J., Weaver D.R., Kolakowski L.F., Reppert S.M. (1997). Two period homologs: Circadian expression and photic regulation in the suprachiasmatic nuclei. Neuron.

[8] Shearman L.P., Sriram S., Weaver D.R., Maywood E.S., Chaves I., Zheng B., Kume K., Lee C.C., Van Der Horst G.T.J., Hastings M.H. (2000). Interacting molecular loops in the mammalian circadian clock. Science.

[9] Zheng B., Larkin D.W., Albrecht U., Sun Z.S., Sage M., Eichele G., Lee C.C., Bradley A. (1999). The mPer2 gene encodes a functional component of the mammalian circadian clock. Nature.

[10] Zheng B., Albrecht U., Kaasik K., Sage M., Lu W., Vaishnav S., Li Q., Sun Z.S., Eichele G., Bradley A. (2001). Nonredundant roles of the mPer1 and mPer2 genes in the mammalian circadian clock. Cell.

[11] Vitaterna M.H., Selby C.P., Todo T., Niwa H., Thompson C., Fruechte E.M., Hitomi K., Thresher R.J., Ishikawa T., Miyazaki J. (1999). Differential regulation of mammalian Period genes and circadian rhythmicity by cryptochromes 1 and 2. Proc. Natl. Acad. Sci. USA.

[12] Aton S.J., Colwell C.S., Harmar A.J., Waschek J., Herzog E.D. (2005). Vasoactive intestinal polypeptide mediates circadian rhythmicity and synchrony in mammalian clock neurons. Nat. Neurosci..

[13] Maywood E.S., Chesham J.E., O’Brien J.A., Hastings M.H. (2011). A diversity of paracrine signals sustains molecular circadian cycling in suprachiasmatic nucleus circuits. Proc. Natl. Acad. Sci. USA.

[14] Mieda M., Ono D., Hasegawa E., Okamoto H., Honma K.-I., Honma S., Sakurai T. (2015). Cellular clocks in AVP neurons of the SCN are critical for interneuronal coupling regulating circadian behavior rhythm. Neuron.

[15] Albrecht U., Zheng B., Larkin D., Sun Z.S., Lee C.C. (2001). mPer1 and mPer2 Are Essential for Normal Resetting of the Circadian Clock. J. Biol. Rhythms.

[16] Nakamura W., Yamazaki S., Takasu N.N., Mishima K., Block G.D. (2005). Differential response of Period 1 expression within the suprachiasmatic nucleus. J. Neurosci..

[17] Wen S., Ma D., Zhao M., Xie L., Wu Q., Gou L., Zhu C., Fan Y., Wang H., Yan J. (2020). Spatiotemporal single-cell analysis of gene expression in the mouse suprachiasmatic nucleus. Nat. Neurosci..

[18] Brancaccio M., Patton A.P., Chesham J.E., Maywood E.S., Hastings M.H. (2017). Astrocytes Control Circadian Timekeeping in the Suprachiasmatic Nucleus via Glutamatergic Signaling. Neuron.

[19] Todd W.D., Venner A., Anaclet C., Broadhurst R.Y., De Luca R., Bandaru S.S., Issokson L., Hablitz L.M., Cravetchi O., Arrigoni E. (2020). Suprachiasmatic VIP neurons are required for normal circadian rhythmicity and comprised of molecularly distinct subpopulations. Nat. Commun..

[20] Abrahamson E.E., Moore R.Y. (2001). Suprachiasmatic nucleus in the mouse: Retinal innervation, intrinsic organization and efferent projections. Brain Res..

[21] Antle M.C., Silver R. (2005). Orchestrating time: Arrangements of the brain circadian clock. Trends Neurosci..

[22] Cheng M.Y., Bullock C.M., Li C., Lee A.G., Bermak J.C., Belluzzi J., Weaver D.R., Leslie F.M., Zhou Q.-Y. (2002). Prokineticin 2 transmits the behavioural circadian rhythm of the suprachiasmatic nucleus. Nature.

[23] Clark D.D., Gorman M.R., Hatori M., Meadows J.D., Panda S., Mellon P.L. (2013). Aberrant Development of the Suprachiasmatic Nucleus and Circadian Rhythms in Mice Lacking the Homeodomain Protein Six6. J. Biol. Rhythms.

[24] Roy A., de Melo J., Chaturvedi D., Thein T., Cabrera-Socorro A., Houart C., Meyer G., Blackshaw S., Tole S. (2013). LHX2 is necessary for the maintenance of optic identity and for the progression of optic morphogenesis. J. Neurosci..

[25] VanDunk C., Hunter L.A., Gray P.A. (2011). Development, Maturation, and Necessity of Transcription Factors in the Mouse Suprachiasmatic Nucleus. J. Neurosci..

[26] Newman E.A., Kim D.W., Wan J., Wang J., Qian J., Blackshaw S. (2018). Foxd1 is required for terminal differentiation of anterior hypothalamic neuronal subtypes. Dev. Biol..

[27] Pandolfi E.C., Breuer J.A., Nguyen Huu V.A., Talluri T., Nguyen D., Lee J.S., Hu R., Bharti K., Skowronska-Krawczyk D., Gorman M.R. (2020). The Homeodomain Transcription Factors Vax1 and Six6 Are Required for SCN Development and Function. Mol. Neurobiol..

[28] Bedont J.L., LeGates T.A., Slat E.A., Byerly M.S., Wang H., Hu J., Rupp A.C., Qian J., Wong G.W., Herzog E.D. (2014). Lhx1 Controls Terminal Differentiation and Circadian Function of the Suprachiasmatic Nucleus. Cell Rep..

[29] Zappone M.V., Galli R., Catena R., Meani N., De Biasi S., Mattei E., Tiveron C., Vescovi A.L., Lovell-Badge R., Ottolenghi S. (2000). Sox2 regulatory sequences direct expression of a β-geo transgene to telencephalic neural stem cells and precursors of the mouse embryo, revealing regionalization of gene expression in CNS stem cells. Development.

[30] Hoefflin S., Carter D.A. (2014). Neuronal expression of SOX2 is enriched in specific hypothalamic cell groups. J. Chem. Neuroanat..

[31] Mercurio S., Serra L., Motta A., Gesuita L., Sanchez-Arrones L., Inverardi F., Foglio B., Barone C., Kaimakis P., Martynoga B. (2019). Sox2 Acts in Thalamic Neurons to Control the Development of Retina-Thalamus-Cortex Connectivity. iScience.

[32] Ferri A., Favaro R., Beccari L., Bertolini J., Mercurio S., Nieto-Lopez F., Verzeroli C., La Regina F., De Pietri Tonelli D., Ottolenghi S. (2013). Sox2 is required for embryonic development of the ventral telencephalon through the activation of the ventral determinants Nkx2.1 and Shh. Development.

[33] Favaro R., Valotta M., Ferri A.L.M., Latorre E., Mariani J., Giachino C., Lancini C., Tosetti V., Ottolenghi S., Taylor V. (2009). Hippocampal development and neural stem cell maintenance require Sox2-dependent regulation of Shh. Nat. Neurosci..

[34] Cavallaro M., Mariani J., Lancini C., Latorre E., Caccia R., Gullo F., Valotta M., DeBiasi S., Spinardi L., Ronchi A. (2008). Impaired generation of mature neurons by neural stem cells from hypomorphic Sox2 mutants. Development.

[35] Ferri A.L.M., Cavallaro M., Braida D., Di Cristofano A., Canta A., Vezzani A., Ottolenghi S., Pandolfi P.P., Sala M., DeBiasi S. (2004). Sox2 deficiency causes neurodegeneration and impaired neurogenesis in the adult mouse brain. Development.

[36] Kelberman D., Rizzoti K., Avilion A.A., Bitner-Glindzicz M., Cianfarani S., Collins J., Chong W.K., Kirk J.M.W., Achermann J.C., Ross R. (2006). Mutations within Sox2/SOX2 are associated with abnormalities in the hypothalamo-pituitary-gonadal axis in mice and humans. J. Clin. Investig..

[37] Cheng A.H., Bouchard-Cannon P., Hegazi S., Lowden C., Fung S.W., Chiang C.K., Ness R.W., Cheng H.Y.M. (2019). SOX2-Dependent Transcription in Clock Neurons Promotes the Robustness of the Central Circadian Pacemaker. Cell Rep..

[38] Cheng A.H., Bouchard-Cannon P., Ness R.W., Cheng H.Y.M. (2019). RNA-sequencing data highlighting the time-of-day-dependent transcriptome of the central circadian pacemaker in Sox2-deficient mice. Data Br..

[39] Srinivas S., Watanabe T., Lin C.S., William C.M., Tanabe Y., Jessell T.M., Costantini F. (2001). Cre reporter strains produced by targeted insertion of EYFP and ECFP into the ROSA26 locus. BMC Dev. Biol..

[40] Kabrita C.S., Davis F.C. (2008). Development of the mouse suprachiasmatic nucleus: Determination of time of cell origin and spatial arrangements within the nucleus. Brain Res..

[41] Achim K., Salminen M., Partanen J. (2014). Mechanisms regulating GABAergic neuron development. Cell. Mol. Life Sci..

[42] Ahern T.H., Krug S., Carr A.V., Murray E.K., Fitzpatrick E., Bengston L., McCutcheon J., De Vries G.J., Forger N.G. (2013). Cell death atlas of the postnatal mouse ventral forebrain and hypothalamus: Effects of age and sex. J. Comp. Neurol..

[43] McNeill D.S., Sheely C.J., Ecker J.L., Badea T.C., Morhardt D., Guido W., Hattar S. (2011). Development of melanopsin-based irradiance detecting circuitry. Neural Dev..

[44] Lodato M.A., Ng C.W., Wamstad J.A., Cheng A.W., Thai K.K., Fraenkel E., Jaenisch R., Boyer L.A. (2013). SOX2 Co-Occupies Distal Enhancer Elements with Distinct POU Factors in ESCs and NPCs to Specify Cell State. PLoS Genet..

[45] Gascón S., Murenu E., Masserdotti G., Ortega F., Russo G.L., Petrik D., Deshpande A., Heinrich C., Karow M., Robertson S.P. (2016). Identification and Successful Negotiation of a Metabolic Checkpoint in Direct Neuronal Reprogramming. Cell Stem Cell.

[46] Hyodo S., Yamada C., Takezawa T., Urano A. (1992). Expression of provasopressin gene during ontogeny in the hypothalamus of developing mice. Neuroscience.

[47] Jing X., Ratty A.K., Murphy D. (1998). Ontogeny of the vasopressin and oxytocin RNAs in the mouse hypothalamus. Neurosci. Res..

[48] Ban Y., Shigeyoshi Y., Okamura H. (1997). Development of vasoactive intestinal peptide mRNA rhythm in the rat suprachiasmatic nucleus. J. Neurosci..

[49] Gross-Thebing T., Paksa A., Raz E. (2014). Simultaneous high-resolution detection of multiple transcripts combined with localization of proteins in whole-mount embryos. BMC Biol..

[50] Wang F., Flanagan J., Su N., Wang L.-C., Bui S., Nielson A., Wu X., Vo H.-T., Ma X.-J., Luo Y. (2012). RNAscope: A novel in situ RNA analysis platform for formalin-fixed, paraffin-embedded tissues. J. Mol. Diagn..

[51] Boehler N.A., Fung S.W., Hegazi S., Cheng A.H., Cheng H.M. (2021). Sox2 Ablation in the Suprachiasmatic Nucleus Perturbs Anxiety- and Depressive-like Behaviors. Neurol. Int..

